# Tocilizumab and Desensitization in Kidney Transplant Candidates: Personal Experience and Literature Review

**DOI:** 10.3390/jcm10194359

**Published:** 2021-09-24

**Authors:** Jules Weinhard, Johan Noble, Thomas Jouve, Paolo Malvezzi, Lionel Rostaing

**Affiliations:** 1 Service de Néphrologie, Hémodialyse, Aphérèses, et Transplantation Rénale, CHU Grenoble-Alpes, 38700 Grenoble, France; jweinhard@chu-grenoble.fr (J.W.); jnoble@chu-grenoble.fr (J.N.); tjouve@chu-grenoble.fr (T.J.); pmalvezzi@chu-grenoble.fr (P.M.); 2 Faculté de Médecine, Université Grenoble-Alpes, 38700 Grenoble, France

**Keywords:** tocilizumab, clazakizumab, desensitization, kidney transplantation, anti-HLA alloantibody

## Abstract

Desensitization (DES) allows kidney transplantation for highly HLA-sensitized subjects. Due to the central role of IL-6 in the immunological response, tocilizumab may improve DES efficacy. Thus, we conducted a PubMed systematic review using the MeSH terms tocilizumab, interleukin-6, kidney transplantation, and desensitization. Tocilizumab (TCZ) was first studied for DES as the second-line treatment after failure of a standard DES protocol (SP) (apheresis, rituximab +/− IVIg). Although TCZ (as a monotherapy) attenuated anti-HLA antibody rates, it did not permit transplantation. However, lymphocyte immuno-phenotyping has shown that TCZ hinders B-cell maturation and thus could improve the long-term efficacy of DES by limiting anti-HLA rebound and so avoid antibody-mediated rejection. This hypothesis is supported by a recent study where clazakizumab, a monoclonal antibody directed against IL-6, was continued after kidney transplantation in association with an SP. Nine out of ten patients were then eligible for transplantation, and there were no donor-specific antibodies at 6 months post-transplantation. In association with an SP, tocilizumab does not seem to significantly improve kidney-allograft access (short-term efficacy) vs. a SP only. However, it could improve the long-term prognosis of HLA-incompatible transplantation by hindering B-cell maturation and, thereby, avoiding donor-specific antibody rebounds post-transplantation.

## 1. Introduction

Kidney transplantation is recognized as the best replacement therapy for end-stage renal disease in terms of survival and quality of life, but also from a medical and economic standpoint [1,2,3]. However, a shortage of kidney grafts increases time on a waiting list, and human leukocyte antigen (HLA)-sensitized kidney transplant candidates (KTCs) are the most affected. As these subjects have developed anti-HLA antibodies during previous allogeneic exposures due to solid-organ transplantation, pregnancy, or blood transfusion, the number of potentially compatible grafts is limited. To improve access to transplantation, a desensitization (DES) protocol can be undertaken to eliminate anti-HLA antibodies and to block their production.

Initially developed in the context of HLA- and ABO-incompatible living donations, DES is now considered to increase the chances of obtaining a graft from a deceased donor. The current standard protocol (SP) for DES has been empirically developed and validated by studies [4,5,6] that describe combining apheresis (plasma exchange, double-filtration plasmapheresis, semispecific immunoadsorption), rituximab (anti-CD20 monoclonal antibody), conventional triple immunosuppression (anti-calcineurin, mycophenolic acid, and steroids), and possibly intravenous polyvalent immunoglobulin (IVIg).

The results of these DES protocols are primarily supported in the living donor transplant setting, and much less so in the deceased donor setting. Montgomery et al. have shown that HLA-incompatible recipients who underwent a DES protocol and received a graft from a living donor had better survival than highly sensitized patients who remained on dialysis or eventually obtained a compatible graft from a deceased donor [7]. The results from this single-center study were supported by a larger multicenter study confirming a survival benefit at 8 years [8]. Both studies used a US-based population. In contrast, in a UK-based study, Manook et al. showed no survival benefit in highly sensitized patients who received an HLA-incompatible living donor transplant compared with patients remaining on dialysis while waiting for a compatible graft from a deceased donor [9]. This difference between the US and UK studies may be explained by the decreased survival rates of dialysis subjects in the US compared to those in the UK. Nevertheless, the results of Manook et al. can be seen from two different angles: the absence of improved survival by the practice of HLA-incompatible transplantation and the absence of excess mortality in DES protocols, which allow improved access to transplantation for highly HLA-sensitized patients and therefore improved quality of life.

In some patients, these DES protocols do not succeed in decreasing the level of anti-HLA antibodies sufficiently to allow the transplantation of grafts carrying the corresponding HLA specificities. One of the hypotheses explaining this is the absence (with the current protocols) of action on plasma cells, particularly on memory plasma cells. With rituximab, this may seem obvious since it is an anti-CD20 monoclonal antibody, and this CD20 marker is no longer expressed by plasma cells. Perry et al. have shown in vitro that rituximab as well as IVIg and anti-thymocyte globulin (ATG; used as a transplant induction therapy) were not able to induce plasma cell apoptosis or block antibody production [10]. Vo et al. showed that in their cohort of 600 highly sensitized patients who received an IVIg + rituximab desensitization protocol, DES failure was more frequent in patients with a high level of memory plasma cells with anti-HLA specificities [11]. Therefore, the idea arose to use treatments that could better target plasma cells. 

First, bortezomib was studied, as an analogy to the treatment of myeloma (precisely due to the monoclonal proliferation of plasma cells). In vivo, in highly sensitized primates, bortezomib decreased the level of antibody-producing cells and CD38 + CD19 + CD20 plasma cells in the bone marrow. However, it did not decrease the level of donor-specific antibodies (DSAs) [12]. In vivo, bortezomib has been shown to decrease DSA levels in highly sensitized patients, but without achieving cross-match negativity through microlymphocytotoxicity, and therefore without improving access to transplantation [13,14]. Due to a lack of evidence of major efficacy and a poor safety profile, bortezomib has not found its place in the field of DES.

In the challenge of finding a molecule that could better target all facets of the immune response responsible for the production of anti-HLA antibodies, the study of the IL-6 blockade is relevant because this cytokine plays a central role in the regulation of the humoral and cellular immune responses. Therefore, we carried out a literature review of studies that specify the place of the anti-IL-6R monoclonal antibody tocilizumab (TCZ) in DES protocols. We first present the studies that justify a pathophysiological rationale for the use of TCZ, and then detail the recent clinical studies that assess TCZ’s efficacy and safety as a DES treatment.

## 2. Materials and Methods

We performed a systematic review of the literature using PubMed with the following MeSH terms: tocilizumab, interleukin-6, kidney transplantation, desensitization.

## 3. Results and Discussion

### 3.1. Pathophysiological Rationale: IL-6 and the Immune Response

Interleukin-6 (IL-6) is a pleiotropic cytokine whose sites of action are as numerous as the possible therapeutic effects of tocilizumab: impact on hematopoiesis (anemia caused by inflammatory syndrome), osteoclast differentiation (osteoporosis), keratocyte proliferation (psoriasis and skin disorders of the scleroderma), and regulation of the hypothalamo–hypophyseal axis [15]. IL-6’s role in the immune response is also decisive. It is interesting to note that it was the ability of IL-6 to induce antibody production by B cells that led to its discovery in 1985 [16]. It was then called B-cell stimulating factor 2 (BSF-2) or B cell differentiation factor (BCDF). IL-6’s central role in both the innate and adaptive immune response is now well documented.

#### 3.1.1. IL-6 and the Innate Immune Response

In the early phases of the innate immune system response, IL-6 is released by monocytes, macrophages, lymphocytes, endothelial cells, and dendritic cells after recognition of the danger signals sent out by the toll-like receptors(TLRs). These signals can be induced by an aggressive infection (pathogen-associated molecular patterns) or not (danger-associated molecular patterns). IL-6 also stimulates the hepatocyte production of inflammation proteins such as C-reactive protein, serum amyloid A protein, haptoglobin, and fibrinogen.

#### 3.1.2. IL-6 and the Humoral Adaptive Immune Response: B-Cell Response

IL-6 plays a role in the differentiation of a mature B cell into a cell capable of secreting antibodies. It promotes the differentiation of CD4+ T cells into the T follicular helper (Tfh) cells, which release the IL-21 necessary for the differentiation of mature B cells into plasma cells [17,18]. It has been shown that plasmablasts have the capacity to produce IL-6 and thus to generate numerous T follicular helper cells. Moreover, circulating levels of these two cell populations are not only higher in patients with rheumatoid arthritis than in the general population, but they are also lower after initiation of tocilizumab [19]. Furthermore, a study in mutated mice that do not express the IL-6 gene showed a clear decline in the immune B-cell response with decreases of blood IgG1, IgG2a, and IgG3 rates [20]. Thus, it is clear that IL-6 is essential for antibody production through inducing plasma cell differentiation, and it plays a role in the generation and regulation of memory B cells. Indeed, the IL-6 blockade by TCZ in rheumatoid arthritis is associated with a significant decrease in pre-switch (IgM+) and post-switch (IgG+) memory B cells [21,22]. IL-6 has also been shown in vivo in mice to participate in the survival of the long-lived plasma cells nested in the bone marrow [23].

#### 3.1.3. IL-6 and the Cellular Adaptive Immune Response: T-Cell Response

Naive CD4+ T cells differentiate into one or another of these effectors (Th1, Th2, Th17, or Treg), each of which will have a different role, depending on the cytokine environment: IL-12 shifts towards a Th1 profile; IL-4 shifts towards a Th2 profile; TGF-β shifts towards a Treg profile (regulatory T cells) profile; and the association of TGF-β and IL-6 shifts towards a Th17 profile [24]. It has also been shown that the orientation towards Th17 or Treg is ‘mutually exclusive.’ Whereas Treg generation requires the exclusive presence of TGF-β, the presence of IL-6 in association with TGF-β will orient towards a Th17 profile while preventing differentiation into Treg [25]. Yet, while Tregs induce immune-tolerance and decrease autoimmune and alloimmune responses, Th17 is associated with the development of autoimmune and chronic inflammatory diseases. It also seems to be involved in the pathophysiology of acute T-cell-mediated rejection after kidney transplantation and in chronic rejection after cardiac transplantation [24,26,27].

The differentiation of CD8+ T cells into cytotoxic T cells is also promoted by IL-6 [28,29]. In addition, the importance of IL-6 in the differentiation and survival of long-term memory T cells has also been demonstrated [30]. Thus, the use of TCZ as a treatment for desensitization could be relevant because of the centrality of IL-6 in the adaptive immune response on both the humoral and cellular levels.

### 3.2. Tocilizumab and Kidney Transplantation

#### 3.2.1. Tocilizumab

Tocilizumab (TCZ) is a monoclonal antibody that is specifically directed against the IL-6 receptor, either the membrane-bound or the soluble form. It was initially approved for the treatment of rheumatoid arthritis and systemic juvenile idiopathic arthritis. Due to the multiple sites of action of IL-6 and a favorable safety profile in early rheumatology cohorts, tocilizumab has been studied for many other indications over the past 15 years, including refractory Takayasu disease [31], systemic lupus erythematosus [32], giant cell arteritis [33], highly relapsing neuromyelitis optica spectrum disorder [34], acute graft-versus-host disease after hematopoietic stem-cell transplantation [35], and even more recently in severe acute respiratory syndrome coronavirus 2 pneumonia [36].

#### 3.2.2. Tocilizumab and Antibody-Mediated Rejection

In the field of kidney transplantation, TCZ was first studied in antibody-mediated rejection (AMR). Chronic active antibody-mediated rejection (cAMR) is a major therapeutic issue in kidney transplantation, as it is one of the main causes of graft loss. However, no treatment has been proven to be superior. Choi et al. used tocilizumab in 36 patients with cAMR who failed the SOC and obtained encouraging results: there was 91% patient survival (overall survival) and 80% graft survival at 6 years, associated with a decrease in DSAs and stable kidney function at 2 years post-transplantation [37]. The researchers observed four instances of cAMR-related graft loss. These occurred in four patients for whom TCZ had been stopped prematurely (one for medical reasons and three for financial reasons), suggesting a rebound effect related to the accumulation of IL-6 under treatment by blocking its receptor with TCZ [38]. To avoid this, clazakizumab, a monoclonal antibody that directly inhibits IL-6, was tested in a randomized controlled trial in 20 patients with late active or chronic active antibody-mediated rejection [39]. After 12 weeks of treatment, the clazakizumab group had a significant decrease in DSA mean fluorescence intensity and a smaller decline in graft function compared to the placebo group. However, the safety analysis (primary endpoint) showed severe infectious complications in five patients (25%) and complicated colonic diverticulitis in two patients (10%). The encouraging efficacy data must be contrasted with the results from a series of nine patients with SOC-resistant cAMR treated with tocilizumab: compared with a historical cohort on SOC alone, tocilizumab did not significantly improve graft function or reduce histological damage [40]. 

To determine whether the potentially beneficial effect of tocilizumab described by Choi et al. requires prior exposure to other immunosuppressive therapies, Lavacca et al. used tocilizumab as a first-line therapy in 15 kidney transplant patients with severe cAMR (60% with transplant glomerulopathy classified as cg3 according to Banff classification) with satisfactory results. Over a follow-up period of 20.7 months, there was a stabilization of renal function, increased proteinuria, a significant decrease in DSAs, improvement of microvascular inflammation lesions on systematic biopsy at 6 months, and stabilization of other histological lesions [41]. However, data on the use of tocilizumab to treat acute active humoral rejection are limited [42].

Chandran et al. performed a randomized controlled trial that included 30 patients with stable graft function, with subclinical inflammation defined on routine biopsy as moderate interstitial inflammation (Banff classification i or ti 1–2 and t0) [43]. Subjects were randomized between a treatment group (tocilizumab 8 mg/kg monthly, 6 injections) and a placebo group. The authors showed a significant decrease in interstitial inflammation associated with an increased level of Tregs in 62.5% of the subjects treated with tocilizumab versus 21.4% in the control group (*p* = 0.03). Given this study’s results, tocilizumab could be of interest for use in the early stages of graft inflammation and even before the rejection stage.

#### 3.2.3. Tocilizumab and Desensitization

Few studies have focused on the use of TCZ in DES protocols in highly sensitized KTCs. An in vivo study in HLA-incompatible (HLA-A2) skin graft sensitized mice suggested that TCZ not only decreases the level of anti-HLA-A2 antibodies, but also the number of plasma cells producing these antibodies in the bone marrow and spleen [44,45]. In humans, Vo et al. tested TCZ as a second-line DES therapy in 10 highly sensitized KTCs who had failed in the standard desensitization protocol (IVIg + rituximab +/− plasma exchange) [11]. The safety profile (primary endpoint) of the treatment was favorable, and 5 of 10 patients were able to receive a transplant after a mean time of 8.1 months, compared to a mean time of 25 months since the first desensitization attempt. There was no AMR on a routine biopsy conducted at 6 months and no development of DSAs. There was AMR at 12 months in one patient, who nonetheless responded well to treatment. Thus, this small study suggests the value of adding TCZ to the standard DES protocol in the patients who are the most difficult to desensitize (see Table 1).

In order to assess the efficacy and safety of a DES protocol based exclusively on tocilizumab Daligault and our team conducted a single-center, non-randomized study in 14 highly sensitized candidates awaiting kidney transplantation who had not previously received any other DES treatment [46]. While having a favorable safety profile (only one serious adverse event of an infectious nature), there was also a significant decrease in the MFI of the immunodominant anti-HLA antibody and in the number of antibodies with MFI > 10,000. However, this decrease was not clinically significant as the MFIs remained at levels incompatible with transplantation, and only one patient could receive a transplant. In contrast, when 11 out of the 14 patients were started on a standard DES protocol (apheresis, rituximab, triple immunosuppression (tacrolimus, mycophenolic acid, steroids)) following tocilizumab therapy, 8 subsequently became eligible for a transplant. Transplantation was performed, on average, 9.6 months after the start of TCZ, whereas waiting times from the date of last enrollment to the start of TCZ averaged 90.2 months.

In comparison, Noble and our team recently published the results of the standard DES protocol used at the University Hospital of Grenoble (apheresis; rituximab; immunosuppression with tacrolimus, mycophenolate mofetil, and steroids) between 2016 and 2020. Forty-five subjects were treated (18 with a living donor, 27 from a deceased donor), including the 13 patients in the Daligault et al. study who had previously benefited from tocilizumab [48]. With the removal of those patients from the sample, 32 patients were treated, 29 of whom were able to undergo transplantation (91%). These studies (Daligault et al. and Noble et al.) therefore suggest that, in terms of improving transplantation access (which could be seen as the ‘short-term efficacy’ of DES), TCZ alone is not sufficient, and the addition of TCZ upstream of a standard DES protocol does not seem to do any better than the standard protocol alone.

However, TCZ may be of interest due to its longer-term effects. Using a cohort of patients in Grenoble treated with TCZ alone, Jouve and our team used flow cytometry to characterize the lymphocyte profiles of these subjects in comparison with highly sensitized patients on dialysis and with healthy subjects [47]. The research showed that TCZ could inhibit B-cell maturation, as evidenced by the significant decrease in post-germinative B cells, plasma cells, and plasmablasts. By hindering B-cell maturation, it is possible that TCZ in association with the standard DES protocol limits the rebound of anti-HLA antibodies after transplantation and thus improves the long-term outcome of DES protocols. Rather than improving access to transplantation (‘short-term efficacy’), tocilizumab would therefore mainly improve long-term graft survival by limiting the risk of post-transplant anti-HLA rebound and, therefore, AMR. 

Therefore, the question could arise as to the value of continuing tocilizumab after HLA-incompatible transplantation. To investigate this, a study was conducted using clazakizumab, a monoclonal antibody directed directly against IL-6 and studied in rheumatoid arthritis (not yet marketed) [49]. Vo et al. used clazakizumab as a DES therapy in 10 highly sensitized KTCs receiving IVIg and plasma exchange in parallel. While having a favorable safety profile, a significant decrease in anti-HLA antibodies was observed between the beginning and end of the treatment in 9 out of 10 patients, all of whom could then undergo transplantation. Clazakizumab was continued after transplantation and none of the patients had a DSA at 6 months post-transplantation. In addition, these patients had a significant increase of FoxP3-expressing Tregs at 6 months post-transplantation, suggesting that clazakizumab could orient the T-cell response towards a tolerogenic profile [50]. Moreover, Jouve et al. noted no significant increase in this lymphocyte population with tocilizumab [47].

## 4. Conclusions

Continued research into new treatments and DES procedures is a major challenge towards improving access to transplantation for highly sensitized subjects while guaranteeing not only safety, but also efficacy. The efficacy of DES has to be judged at the individual level (access to transplantation, long-term graft function) and at the collective level (respect for the principle of utility) Desensitization is sufficiently effective such that offering a graft to a highly sensitized subject does not shorten the graft’s lifespan compared to that expected in a non-sensitized subject. Tocilizumab is a relevant candidate because of the centrality of IL-6 in the regulation of a cellular and humoral immune response. When used as a single agent without any other DES treatment, tocilizumab reduces anti-HLA antibodies, but this is not sufficient to allow transplantation. However, the TCZ-induced blockage of B-cell maturation suggests that its addition to the standard DES protocol could improve the long-term outcome of HLA-incompatible transplants by decreasing post-transplant antibody rebound. The continuation of TCZ after transplantation could then be justified to decrease the risk of humoral rejection. The efficacy and safety of such a procedure remain to be demonstrated in a study that compares this strategy to the standard DES protocol.

## Figures and Tables

**Table 1:** Key safety and efficacy studies using tocilizumab as a desensitization therapy.

Study	Design/Population	Intervention	Outcomes	Main Results
Kim et al. [44] Transplantation 2014	Pre-clinical study, in vivo HLA-incompatible (HLA-A2) skin graft sensitized mice	TCZ intraperitoneal (10–30 mg/kg ×3/week, during 4 weeks) vs. placebo	MFI anti-HLA-A2 Ab Rates of - T fh - T fh 17 - T reg - long-term PC	↘ MFI anti-HLA-A2 Ab (*p* = 0.0076) ↘ rates: - T fh - T fh 17 - long-term PC ↗ T reg
Vo et al. [11] Transplantation 2015	Phase I/II monocentric, uncontrolled study 10 HS patients After failure of DES with RTX + IVIg +/− PE	TCZ IV 8 mg/kg at J15 then 1/month during 6 months + IVIg at J0 and J15 (2 g/kg)	Efficacy: - % of patients receiving a transplant - Rejection at M6 biopsy - DSA at M6 Safety	5/10 patients received a transplant (mean delay of 8.1 months post-1st TCZ) At M6: no DSA, no AMR At M12: 1 AMR (good response to treatment); no graft loss 2 serious AEs: - Acute pulmonary edema (dialysis insufficiency) with epilepsy (not related to TCZ) - Colonic diverticulitis with perforation (possibly related to TCZ)
Daligault et al. [46] Transplantation Direct 2021	Phase II monocentric, uncontrolled study HS patients; First DES attempt	TCZ IV 8 mg/kg (1/month; during ≥6 months) No other prior or concurrent DES procedures	Efficacy: - MFI of anti-HLA immunodominant Ab - Number of anti-HLA Ab with MFI > 10,000 - % of patients received a transplant Safety	↘: - MFI of anti-HLA immunodominant Ab (*p* < 0.05) - Number of anti-HLA Ab with MFI > 10 000 (*p* < 0.05) Not clinically relevant: only 1 patient received a transplant 1 serious AE: spondylodiscitis
Jouve et al. [47] AJT 2021	Monocentric, controlled, non-randomized study HS patients (first DES attempt) Control groups: - HS patients remaining on dialysis without DES attempt - Healthy subjects	TCZ IV 8 mg/kg (1/month; during ≥6 months) No other prior or concurrent DES procedures	Rates evolution of: - T fh 1 ; T fh 2 ; T fh 17 ; T reg - Plasmablasts, plasma-cells, B memory cells Evolution of anti-HLA Ab MFI	T population: No significant change: T fh 1; T fh 2; T fh 17; T reg B population Blocking of maturation ↘: - Post-germinative B-cells - Plasma-blasts - Plasma-cells Anti-HLA Ab MFI: Same observation as Daligault et al. (same cohort)

Abbreviations: Ab: antibody; AE: adverse event; AMR: antibody-mediated rejection; DES: desensitization; IV: intravenous; IVIG: intravenous polyvalent immunoglobulin; HS: highly sensitized; MFI: mean fluorescence intensity; PE: plasma exchange; PC: plasma cells; RTX: rituximab; T fh: T follicular helper cells; T reg: T regulatory cells; ↘ : decrease ; ↗ : increase.
